# Reduced IκB-α Protein Levels in Peripheral Blood Cells of Patients with Multiple Sclerosis—A Possible Cause of Constitutive NF-κB Activation

**DOI:** 10.3390/jcm9082534

**Published:** 2020-08-06

**Authors:** Jun Yan, Pamela A. McCombe, Michael P. Pender, Judith M. Greer

**Affiliations:** 1 UQ Centre for Clinical Research, The University of Queensland Centre for Clinical Research, Brisbane, QLD 4029, Australia; j.yan@uq.edu.au (J.Y.); Pamela.McCombe@uq.edu.au (P.A.M.); 2 Wesley Medical Research, The Wesley Hospital, Auchenflower, QLD 4066, Australia; 3 Faculty of Medicine, The University of Queensland, Brisbane, QLD 4029, Australia; m.pender@uq.edu.au; 4 Department of Neurology, Royal Brisbane and Women’s Hospital, Brisbane, QLD 4029, Australia

**Keywords:** multiple sclerosis, IκB-α, NF-κB, IKK

## Abstract

NF-κB signaling pathways are dysregulated in both the central nervous system (CNS) and peripheral blood cells in multiple sclerosis (MS), but the cause of this is unknown. We have recently reported that peripheral blood mononuclear cells (PBMC) of patients with MS have increased constitutive activation and translocation of the transcription factor NF-κB to the nucleus compared to healthy subjects. NF-κB can be activated through either canonical or non-canonical pathways. In the canonical pathway, activation of NF-κB is normally negatively regulated by the inhibitor IκB. We therefore hypothesized that the increased activation of NF-κB could be caused by reduced IκB-α in the cells of patients with MS, possibly due to increased activity of the IκB kinase (IKK) complex, which regulates IκB-α. Alternatively, changes to the activity of key molecules in the non-canonical pathway, such as IKKα, could also lead to increased NF-κB activation. We therefore used Western blotting to detect IκB-α levels and ELISA to investigate NF-κB DNA binding activity and phosphorylation of IKKα and IKKβ in samples from PBMC of MS patients and controls. The level of full-length IκB-α protein in the cytosolic fraction of PBMC of MS patients was significantly reduced compared to healthy subjects, with significantly more evidence of multiple low molecular weight putative degradation products of IκB-α present in MS patients compared to healthy subjects. Conversely, the level of NF-κB DNA binding activity was increased in whole cell lysates from MS patients. Both IKKα and IKKβ showed increased overall activity in MS compared to healthy subjects, although not all of the MS patients showed increased activity compared to the healthy subjects, suggesting that there may be several different mechanisms underlying the constitutive activation of NF-κB in MS. Taken together, these findings suggest that there may be multiple points at which the NF-κB pathway is dysregulated in MS and that decreased levels of the full-length IκB-α protein are a major component in this.

## 1. Introduction

Multiple sclerosis (MS) is a common neurological disorder characterized by inflammation, demyelination and axonal damage in the central nervous system (CNS) and is considered to be an autoimmune disease [1,2]. The risk of developing MS can be attributed to both genetic and environmental factors. Large genome-wide studies have identified more than 200 discrete genomic loci in addition to the major histocompatibility complex associated with susceptibility to MS [3,4,5]. However, the etiology and pathogenesis of MS are still not fully understood. In MS there is both inflammation that leads to the formation of demyelinated lesions in the brain and spinal cord and neurodegeneration that leads to disease progression [6]. Accumulated research has revealed that, in general, inflammation is regulated to a great extent by the NF-κB signaling pathway [7,8].

NF-κB is a family of 5 structurally related transcription factors (p50/NF-κB1, p52/NF-κB2, p65/ RelA, RelB and c-Rel) that control many cellular functions, in particular, immune responses and apoptosis. Normally NF-κB hetero- or homodimers are sequestered in the cytoplasm of the cells by inhibitory proteins that are characterized by the presence of ankyrin repeats, the best-known of which is the inhibitor of NF-κB (IκB) family [9], but upon activation, they rapidly translocate to the nucleus and mediate transcription of a wide range of target genes. In MS, dysregulated NF-κB activation has been observed in both peripheral blood immune cells and within active lesions in the CNS [10,11,12,13,14,15,16], and multiple genes within or regulated by the NF-κB pathway have been linked to the development of MS [17]. Therefore, it is important to improve our understanding of how NF-κB is dysregulated in MS, and what the implications of dysregulated NF-κB signaling are for therapeutic strategies in MS [14].

**Figure 1:**
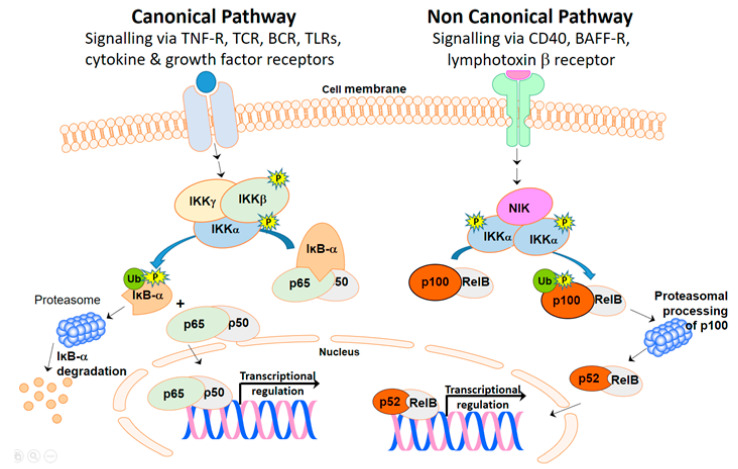
The canonical NF-κB pathway can be activated via multiple different ligand/receptor interactions. Signaling through these receptors eventually converges on the IKK complex (made up of IKKα, IKKβ and IKKγ) where it causes phosphorylation and activation. Activated IKK triggers the phosphorylation and subsequent degradation of the IκB-α via the ubiquitin-proteasome, releasing the bound NF-κB dimer (p65/p50 dimer is shown in this diagram). The NF-κB dimer can then move into the nucleus and bind to DNA to cause transcriptional regulation. In the non-canonical pathway, signaling via a more limited set of receptors converges on NIK, which then binds to and phosphorylates a dimer of IKKα. Phosphorylated IKKα causes the phosphorylation and ubiquitination of p100, which is complexed to RelB. p100 is cleaved through proteasomal degradation to produce a p52 NFκB subunit complexed with RelB to form the active NF-κB heterodimer, which can then move into the nucleus and bind to DNA to cause transcriptional regulation. Abbreviations: TNFR, Tumor Necrosis Factor Receptor; TCR, T cell receptor; BCR, B cell receptor; TLRs, toll-like receptors; BAFF-R, B-cell activating factor receptor; Ub, ubiquitin.

NF-κB can be activated through canonical and non-canonical pathways (Figure 1). The canonical pathway is activated via the interaction of specific ligands with a wide range of receptors, including T cell- and B cell-receptors, cytokine receptors, pattern recognition receptors, and tumor necrosis factor receptor (TNFR) superfamily members. Activation of the canonical pathway (left hand side of Figure 1) eventually converges on an IκB kinase (IKK) complex, which is comprised of two kinases (IKKα and IKKβ) and a regulatory subunit, NEMO/IKKγ [7,18]. When the IKK complex is activated, IKKα generally only makes a small contribution towards the overall kinase activity [19], and it is primarily IKKβ that triggers the phosphorylation and subsequent degradation of the IκBs via the ubiquitin-proteasome pathway. The best-studied and most important of the IκBs is IκB-α, which primarily binds the NF-κB heterodimers p50/p65 and p50/c-Rel [20].

The noncanonical NF-κB pathway (right hand side of Figure 1) is activated by a more limited group of stimuli that bind to a subset of the TNF receptor superfamily members, including lymphotoxin β receptor, B-cell activating factor (BAFF) receptor, receptor activator for NF-κB (RANK), TNFR2, and CD40, and appears to cooperate with the canonical pathway to regulate specific functions of the adaptive immune system [7,21]. A central player in the activation of the noncanonical NF-κB pathway is NF-κB kinase (NIK), which activates and cooperates with IKKα to phosphorylate a precursor of p52/NF-κB2, p100. p100 serves an IκB-like function, due to the presence of ankyrin repeats in its C-terminal portion, but is degraded following phosphorylation by IKKα, leading to generation of mature p52 and nuclear translocation of a p52/RelB dimer [21].

In the experimental autoimmune encephalomyelitis animal model of MS, both the canonical and noncanonical pathways of NF-κB activation appear to be involved and to work co-operatively in development of disease [22,23,24,25]. Recently, we have described constitutive translocation of p65/RelA to the nucleus in innate and adaptive immune cells from the blood of people with MS [16], but it is not yet known if other parts of the NF-κB pathway are also dysregulated. In this paper, the aim was to investigate levels of molecules important in the canonical and non-canonical NF-κB pathways. Therefore, we have compared the protein levels of IκB-α in the cytoplasm of peripheral blood cells from MS patients and healthy individuals, the NF-κB DNA binding potential of nuclear and whole cell lysates from MS patients and healthy controls and finally have compared the levels of activation of IKKα and IKKβ. The results suggest dysregulation of both the canonical and non-canonical pathways in MS patients.

## 2. Experimental Section

### 2.1. Patients and Controls

Blood samples from a total of 39 MS patients and 32 healthy individuals were collected for this study. Patients with MS were recruited through the MS Clinic, Royal Brisbane and Women’s Hospital, Brisbane, Australia. The MS group included 21 relapsing-remitting MS (RR-MS) patients, 10 secondary progressive MS (SP-MS) patients, and 8 primary progressive MS (PP-MS) patients. All patients with MS met the 2010 revised McDonald criteria for MS [26], apart from 2 of the PP-MS patients. Those two PP-MS patients were diagnosed on clinical and cerebrospinal fluid (CSF) criteria and met the laboratory-supported MS definition of Poser [27]. None of the MS patients had been treated with corticosteroids or immunomodulatory therapy in the 3 months prior to blood collection. Healthy controls were recruited from hospital and university staff. All participants were from Australia or New Zealand and were of Caucasian ethnic background. Informed consent was obtained prior to blood collection. Demographic details of patients and controls are shown in Table 1. This study was approved by the Human Research Ethics Committees of the Royal Brisbane and Women’s Hospital (HREC/11/QRBW/92) and The University of Queensland (201605364).

### 2.2. Collection of Blood, Separation of Peripheral Blood Mononuclear Cells (PBMC) and Preparation of PBMC Fractions.

Twenty-five ml of peripheral blood was collected from each of the MS patients and healthy controls. PBMC were isolated via centrifugation through LymphoSep Medium (MP Biomedicals, Sydney, NSW, Australia). After 3 washes in phosphate buffered saline (PBS), aliquots of 10^7^ PBMC were immediately snap frozen in liquid nitrogen for the protein studies.

Cytoplasmic and nuclear fractions of PBMC were prepared using a NE-PER^TM^ Nuclear and Cytoplasmic Extraction kit (Thermo-Fisher, Waltham, MA, USA), as per the manufacturer’s protocol. Whole cell lysates of PBMC were prepared using a PathScan Sandwich ELISA lysis buffer (Cell Signaling Technology, Danvers, MA, USA). The protein concentration of each fraction was determined using a Quick Start^TM^ Bradford Protein Assay kit (Bio-Rad, Gladesville, NSW, Australia) with bovine serum albumin standards. In Western blots (see 2.3), anti-PCNA antibody (ab152112, Abcam, Cambridge, UK) and anti-PKCα antibody (ab4124, Abcam) were used to ensure that the cytosolic fractions did not contain nuclear material and that the nuclear fraction did not contain cytosolic proteins, respectively (Supplementary Figure S1).

### 2.3. Western Blotting to Detect IĸB-α and p52

For detection of IκB-α, cytosolic protein fractions (30 µg of each) were separated by SDS polyacrylamide gel electrophoresis (SDS-PAGE) on 10% gels and transferred to Amersham Hybond polyvinylidene difluoride (PVDF) membranes (GE Healthcare Life Sciences, Parramatta, NSW, Australia). The membranes were blocked for 1 h at room temperature in 5% skimmed milk in Tris-buffered saline plus 0.1% Tween-20 and then incubated with C-terminus anti-IĸB-α antibody (sc-371 (clone C-21), Santa Cruz, Dallas, TX, USA) or a N-terminus anti-IκB-α antibody (Cat # 9242, Cell Signaling Technology). For detection of p52, cytosolic and nuclear fractions were prepared as previously described [16], and fractions were separated by SDS-PAGE and transferred to PVDF membranes as above, with an anti-p52 antibody (sc-848, Santa Cruz) used to detect p52 in the nuclear and cytoplasmic fractions. For detection of both IκB-α and p52, antibody against actin (pan Actin Ab-5, Clone ACTN05; ThermoFisher Scientific, Australia) was used to control for gel loading. After washing, the membranes were incubated with HRP-conjugated secondary antibodies, washed again, and then bands were detected using SuperSignal^TM^ West Pico Plus Chemiluminescent substrate (ThermoFisher). The fluorescence signal of each protein was acquired using a ChemiDoc^TM^ MP Imaging System (BioRad) at set exposure times for each protein. Band density was determined using NIH Image J and was normalized for actin loading. To compare the proportion of full-length versus truncated IκB-α, if no band was present for either one band or another, that band was given a value of 100 pixels (one half the value of the lowest intensity band detected).

### 2.4. Assay for NF-κB p65 DNA Binding Activity

A TransAM^®^ NF-κB Family Transcription Factor Assay Kit (Active Motif, Carlsbad, CA, USA) was used to detect DNA binding of activated NF-κB p65 in nuclear fractions and whole cell lysates. In brief, 5 μg of nuclear protein or 10 µg whole cell lysate in diluent was added in duplicate to wells of an ELISA plate on which the NF-κB family consensus DNA oligonucleotide (5’-GGGACTTTCC-3’) is immobilized. This oligonucleotide binds any activated NF-κB subunits in the nuclear fractions and whole cell lysates. The amount of NF-κB p65 subunit was then detected by addition of a p65-specific detection antibody. A secondary antibody conjugated to horseradish peroxidase (HRP) was then added, followed by 3,3′,5,5′-tetramethylbenzidine (TMB) substrate. After stopping the colorimetric reaction, the absorbance was read at 450 nm (reference wavelength 655 nm) using a Tecan Spark 10M plate reader (Tecan, Port Melbourne, VIC, Australia).

### 2.5. Detection of Phosphorylated (activated) IKKα and IKKβ

Whole cell lysates were tested using PathScan Phospho-IKKα (Ser176/180) and PathScan Phospho-IKKβ (Ser177/181) sandwich ELISA kits (Cell Signaling Technology) to determine the amount of phosphorylated (activated) IKKα and IKKβ in the samples. The manufacturer’s protocol was followed. In brief, 50 µg of whole cell lysate in reaction buffer was added to duplicate wells of ELISA plates coated with a capture antibody specific for the phosphorylated form of IKKα or IKKβ. Following incubation and washing, a detection antibody was added to each well, which was then detected using an HRP-conjugated secondary antibody and a TMB substrate, as described in 2.4.

### 2.6. Statistical Analyses

Data were first assessed to determine if they were normally distributed. As data were not normally distributed, comparisons between groups were assessed by two sided Mann–Whitney U tests using GraphPad Prism 8.0.2. Data were considered to be significant if *P* < 0.05.

## 3. Results

### 3.1. IĸB-α Protein Levels in PBMC from MS Patients and Controls

**Figure 2:**
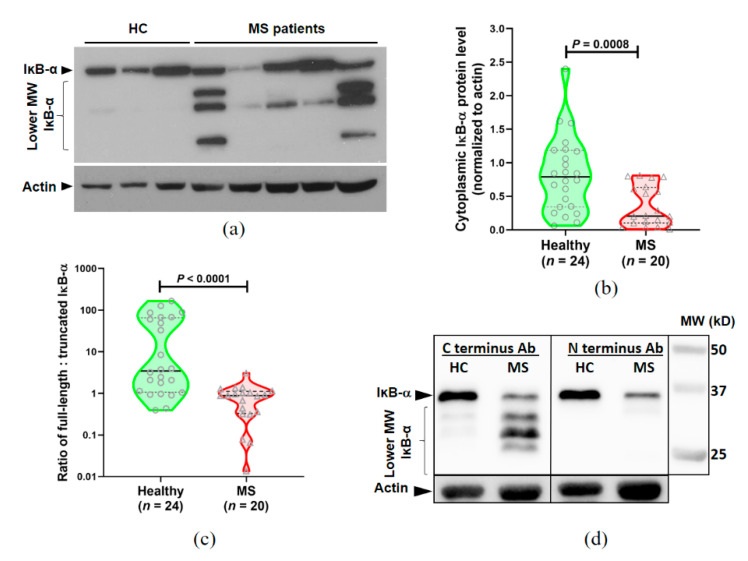
Reduced IκB-α levels in MS. (**a**) Western blot of the cytosolic fraction of PBMC showing Table 36. kD and multiple lower MW bands, particularly in MS patients. (**b**) Violin plots showing the results of the level of full-length (36 kD) IκB-α in the cytosolic fraction of PBMC from healthy individuals or MS patients, normalized to actin. In these violin plots, a solid horizontal line indicates the median, and dotted horizontal lines show the interquartile range. (**c**) Violin plots of the ratio of the full length IκB-α vs the lower MW form in PBMC from healthy individuals or MS patients show a highly significant difference between healthy subjects and patients with MS. (**d**) Representative Western blot showing that the truncated/degraded IκB-α bands could be detected using antibodies specific for the C-terminus but not the N-terminus of IκB-α. For statistical analyses, comparisons between healthy individuals and the MS group were analyzed by two-tailed Mann–Whitney U test.

The protein level of IκB-α in the cytosol of PBMC was determined by Western blotting, in which full length IκB-α can be observed as a band at 36 kD (Figure 2a). In some of the samples, especially those from MS patients, there were also multiple lower MW bands that were labelled with the anti-IκB-α antibody, and which were in the range of 15–30 kD (Figure 2a). They appear to represent degraded fragments or truncated forms of IκB-α that still retain the binding site for the detection antibody.

We first compared the level of full-length IκB-α (normalized to actin) in patients with MS and healthy subjects. MS patients had significantly lower levels of full-length IκB-α than healthy subjects (Figure 2b). A potential limitation of this part of the study is that there was a significant difference in the age of SP-MS and PP-MS patients compared to both the RR-MS patients and healthy controls (see Table 1). Therefore, we determined if the different MS subtypes showed different levels of IκB-α. Compared to controls, all 3 MS subgroups (RR-MS, SP-MS and PP-MS) had significantly less cytoplasmic IκB-α, but the levels did not differ significantly between the MS subtypes, and there was no effect of age (not shown). We also investigated if there was any correlation between the levels of cytoplasmic IκB-α and their disability (EDSS) scores. There was a non-statistically significant trend towards lower levels of cytoplasmic IκB-α in patients with higher EDSS scores (R^2^ = 0.34; *P* = 0.095).

Because some individuals had an increase in the amount of lower MW IκB-α fragments in the Western blots, we next compared the ratio of 36 kD full length IκB-α versus the lower MW bands for healthy subjects and MS patients (Figure 2c). A value greater than 1 indicates that more IκB-α is present as the full-length form, whereas values less than 1 indicate that more of lower MW bands are present. Most (19/24) healthy subjects had more of the full-length form of IκB-α present, whereas the lower MW forms were the predominant form in 13/20 MS patients (Figure 2c). The ratio of full-length to lower MW IκB-α did not differ significantly amongst the different MS subtypes. To investigate these lower MW forms of IκB-α further, duplicate pairs of healthy subject and MS patient samples were run on the same gel, which was then cut in half and blotted with antibodies specific for either the C-terminus or the N-terminus of IκB-α. As shown in Figure 2d, only the C-terminus antibodies were able to detect the lower MW IκB-α, suggesting that the IκB-α is being degraded from the N terminus.

### 3.2. Binding Activity of NFĸB-α p65 in PBMC from MS Patients and Controls

**Figure 3:**
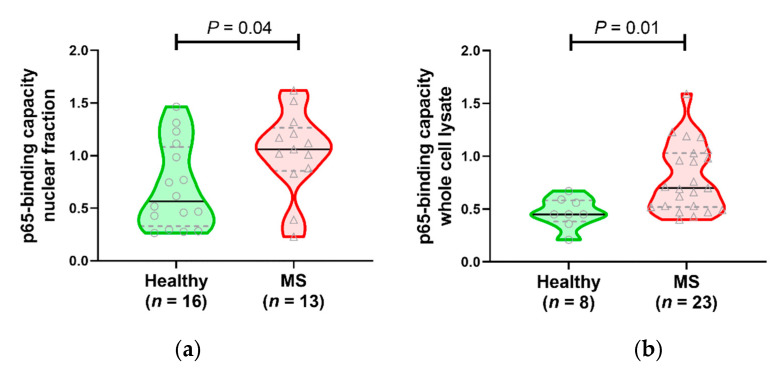
DNA binding activity of p65 from MS patients and healthy subjects. (**a**) p65 binding to the consensus DNA oligonucleotide in cell nuclear extracts was significantly elevated in MS patients compared to healthy individuals. (**b**) In whole cell lysates, the amount of p65 that could bind to the consensus DNA oligonucleotide was also significantly elevated in MS. In these violin plots, a solid line indicates the median, and dotted lines show the interquartile range. Analyzed by two-tailed Mann–Whitney U test.

Next, we evaluated whether the activity of NF-κB p65 subunit (which is very important in the canonical pathway), as measured by its ability to bind to the DNA oligonucleotide 5′-GGGACTTTCC-3′, differed between healthy subjects and patients with MS, since one previous publication has suggested that no differences occur [11]. Nuclear fractions and the whole cell lysates of PBMC were prepared for both healthy subjects and MS patients. Figure 3 shows that NF-κB p65 binding activity is increased in both fractions for some MS patients, although, for many, the binding capacity is within the range seen for healthy subjects, especially in the nuclear fraction.

### 3.3. Activation (Phosphorylation) of IKKα and IKKβ is Increased in MS Patients vs Healthy Subjects

**Figure 4:**
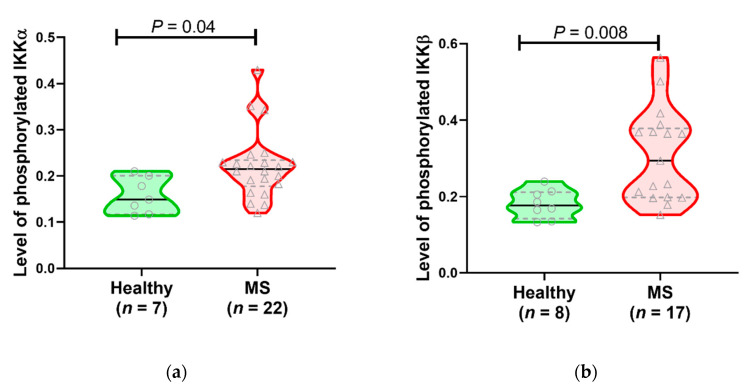
Levels of phosphorylated IKKα and IKKβ are elevated in MS patients compared to healthy subjects. (**a**) Levels of phosphorylated IKKα are increased in some (but not all) MS patients. (**b**) Elevated levels of phosphorylated IKKβ are present in MS patients compared to healthy controls. In these violin plots, a solid line indicates the median, and dotted lines show the interquartile range. Analyzed by two-tailed Mann–Whitney U test.

Phosphorylation of specific serine residues in IKKα (Ser176 and Ser180) and IKKβ (Ser177 and Ser181) is required for their activation in response to cell surface stimulation. Therefore, the phosphorylation at IKKα Ser176/180 and IKK-β Ser177/181 was examined in whole cell lysates of unstimulated PBMC from healthy subjects and MS patients. IKKα phosphorylation was increased in some MS patients compared to healthy subjects, although many of the MS patient values were within the range of values in the healthy subjects, possibly indicating that the non-canonical pathway, which is dependent on phosphorylation of IKKα, is only activated in a fraction of MS patients (Figure 4). For IKKβ, phosphorylation was robust in about half of the MS patients but within the healthy control range for the remainder. These results suggest that increased activation of the canonical pathway (leading to decreased levels of IκB-α and elevated NF-κB p65 DNA binding) may be due to events primarily affecting IKKβ in some MS patients.

### 3.4. Increased transloCation of p52 to the Nucleus in MS Patients

Since IKKα phosphorylation was also somewhat elevated in patients with MS compared to healthy individuals, we also looked at the relative amounts of p52, which is involved in the non-canonical pathway, in the cytoplasm vs the nucleus of 7 healthy individuals and 11 patients with MS. Overall the amount of p52 detectable in samples from all individuals was quite low, and much longer exposure times of Western blots had to be used to detect p52 than for any of the other molecules detected in this study. However, the amount of p52 in the nucleus (as a percentage of the total amount of p52) was significantly increased in the MS group compared to healthy individuals (*P* = 0.009). The range of the percentages of p52 in the nucleus of cells from MS patients varied considerably from one patient to another.

**Figure 5:**
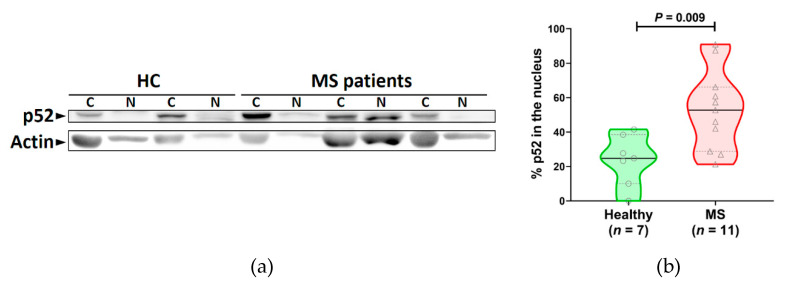
The percentage of total p52 found in the nucleus is significantly increased in MS patients compared to healthy individuals. (**a**) Representative blot of p52 and actin in cytoplasmic (**c**) and nuclear (N) fractions of cells from healthy subjects and MS patients. (**b**) Summary of the percentage of the total amount of p52 in the nucleus. In the violin plots, a solid line indicates the median, and dotted lines show the interquartile range. Analyzed by two-tailed Mann–Whitney U test.

## 4. Discussion

In our previous study, we described increased constitutive activation of the NF-κB p65 subunit, the main subunit relevant to the canonical NF-κB pathway, in peripheral blood cells (particularly T cells and macrophages) of MS patients [16], but the mechanisms underlying these effects were unknown. In the current study, in order to understand what triggers the increased constitutive activation of NF-κB in MS, we looked firstly at levels of the immediate regulator of p65, IκB-α.

As might be expected, based on the elevated levels of p65 translocation into the nucleus in MS [16], levels of the full-length 36 kD IκB-α were significantly decreased in MS patients compared to healthy subjects. However, we also observed that many of the MS patients and a small number of healthy individuals also had multiple smaller sized bands that labelled with an anti-IκB-α antibody which recognizes the C terminus of IκB-α but not with an anti-IκB-α antibody that recognizes the N terminus of the molecule. The low MW bands that labelled with the C terminus antibody do not appear to just be non-specific bands, as they were not present in all patients. We suggest that they are truncated forms or degradation products of IκB-α. The way in which proteins are processed by the proteasome varies from protein to protein, with some being degraded exclusively from either the C terminus or the N terminus and others showing no directional preference: this appears to be determined by the structure of each individual protein domain, with poorly structured domains being more susceptible to degradation [28]. Our results could therefore imply that there is a defect in the structure of IκB-α in MS patients. Interestingly, truncated forms of IκB-α have been reported previously in several pathological conditions. In the first of these, ectodermal dysplasia with immunodeficiency, a c.40G>T mutation in *NFKBIA*, the gene encoding IκB-α, results in a deletion of the N terminus of the IκB-α and production of a 31 kD form of IκB-α [29]. A different mutation in *NFKBIA* has also been associated with decreased IκB-α protein levels and the presence of at least two smaller molecular weight IκB-α bands in gels in patients with glioblastoma multiforme [30]. As noted earlier, IκB-α in the cytoplasm is normally tightly bound to NF-κB, and phosphorylation and ubiquitination of IκB-α are required to release the NF-κB subunit and to commence the degradation of IκB-α in the proteasome [31,32,33]. Free IκB-α is normally very unstable, with an in vivo half-life of around 10 min, and is rapidly degraded via a process that does not require phosphorylation or ubiquitination [31]. However, it has also been reported that certain introduced mutations in *NFKBIA* can slow the degradation process of free IκB-α and stabilize lower molecular weight forms of IκB-α [31,32,33,34]. Our finding of multiple low molecular weight forms of IκB-α in MS patients may indicate that there are specific mutations in *NFKBIA* in MS patients that stabilize the degradation products of IκB-α. It would therefore be of interest in the future to sequence *NFKBIA* from patients with MS to investigate whether they have any mutations that might explain the presence of the smaller size bands of IκB-α in MS patients. One study has reported a genetic variant in the promoter region of *NFKBIA* that appeared to be protective for primary progressive MS [35], but thus far, there have been no other reports of mutations in this gene in MS.

We also investigated the DNA binding capacity of the NF-κB p65 subunit, from both nuclear and whole cell fractions, to bind to a specific target DNA sequence. One previous study using samples from 10 MS patients and 11 healthy individuals reported that there was no difference between the two groups in the DNA binding capacity of nuclear p65 purified from lymphocytes [11]. In the nuclear fractions we tested (Figure 2a), the range of values was very similar for healthy individuals and MS patients, but the MS group was mostly within the higher part of the range, whereas healthy individual values were spread evenly across the range. One possible reason for the differences in our group compared to the previous study is that that study removed macrophages from the PBMC population prior to testing, whereas we did not. Our previous study on NF-κB in MS patients showed that macrophages and T cells were the cell types in which the highest levels of p65 translocation occurred, and therefore, the presence of macrophages in our samples in the current study may be why we found an overall statistically significant higher level of DNA binding of p65 in MS patients than in healthy subjects. In the whole cell fraction, about half of the MS group had levels within the range of the healthy subjects, but the other MS patients were elevated compared to the healthy subjects. The reason for why the binding capacity of p65 from MS patients is significantly higher in the whole cell fraction is not clear; however, it is likely to be influenced by levels of free and bound IκB-α in the cytoplasm.

The levels of phosphorylated (i.e., activated) IKKα and IKKβ were also investigated. There was a robust significant increase in the level of phosphorylated IKKβ in PBMC from MS patients compared to healthy individuals. IKKα phosphorylation was also increased in some MS patients compared to healthy subjects, possibly indicating that the non-canonical pathway, which is dependent on phosphorylation of IKKα, is only activated in a fraction of MS patients. It is possible that the increased activation of IKKs could be due to increased amounts of total IKK protein, which could themselves be due to MS-specific defects at the level of transcription or translation or to increased cell surface receptor binding activity or to altered signaling at any of the molecules upstream of IKK. Irrespective of how the IKKs are hyperphosphorylated in this study, the elevated levels of phosphorylation of IKKβ correlated with the increased activation of IκB-α. Since IKKα phosphorylation can indicate activation of the non-canonical pathway, we also compared the proportion of p52 (which translocates to the nucleus after activation of the non-canonical NF-κB pathway; see Figure 1) in the cytoplasm and nucleus in MS patients and healthy subjects and found that MS patients generally had a higher percentage of their p52 in the nucleus. Overall, however, the amount of p52 detected in both healthy individuals and MS patients was low, suggesting that the non-canonical pathway may be less dominant than the canonical pathway in the altered NF-κB pathway activation seen in MS patients.

In conclusion, we observed a low IκB-α protein level in the cytoplasm of PBMC cells extracted from patients with MS. This is accompanied by increased NF-κB activation through the canonical pathway and increased upstream IKK activity in some patients. Whilst the effects of these changes on expression of proinflammatory molecules that are regulated by the NF-κB signaling pathway, such as IL-1, IL-6 or TNF-α were not assessed in this current study, it is very likely that the increased activation of the NF-κB pathway would result in upregulation of these molecules and of other molecules that are regulated by NF-κB and that are associated with inflammation [7]. Of relevance to MS, these NF-κB-regulated molecules include cytokines, chemokines, adhesion molecules and anti-apoptotic factors that have been reported to be upregulated in MS [36,37,38,39,40,41,42,43,44,45,46,47]. Further investigation of protein interactions and gene mutations/variants in the molecules in the NF-κB pathway, particularly in the canonical pathway, is needed to explain the defects that we have observed in cells from patients with MS. Treatment strategies that normalize and/or stabilize IκB-α may be of therapeutic benefit for MS patients.

## Figures and Tables

**Table 1:** Demographics of participants in the study.

Subjects	Number	% Female	Age (Mean and Range)	EDSS (Mean and Range)
Whole study				
Total MS	39	76.9	45.8 (25–76) ^§^	3.9 (0–6.5)
* RR-MS	21	81.0	37.9 (25–51)	2.7 (0–5.0)
SP-MS	10	80.0	56.3 (48–72) ^§^	5.1 (2.5–6.5)
PP-MS	8	62.5	53.5 (40–76) ^§^	4.8 (2.0–6.5)
Healthy controls	32	90.6	35.5 (22-55)	N/A
Samples used in Figure 2				
Total MS	20	70.0	48.6 (30–76) ^§^	3.9 (1.0–6.5)
RR-MS	8	87.5	39.8 (30–47)	2.7 (1.0–3.5)
SP-MS	6	83.3	54.2 (48–67) ^§^	4.6 (2.5–6.0)
PP-MS	6	50.0	54.6 (40–76) ^§^	4.7 (2.0–6.5)
Healthy controls	24	86.4	37.1 (22-55)	N/A
Samples used in Figure 3				
Total MS	29	79.3	44.8 (25–76)	3.7 (0–6.5)
RR-MS	18	77.8	37.8 (25–50)	2.4 (0–3.5)
SP-MS	7	85.7	56.7 (48–72) ^§^	5.4 (3.5–6.5)
PP-MS	4	75.0	55.3 (40–76) ^§^	5.8 (5.0–6.5)
Healthy controls	18	94.4	37.8 (23–55)	N/A
Samples used in Figure 4				
Total MS	25	84.0	43.6 (25–76)	3.5 (0–6.5)
RR-MS	18	77.8	37.4 (25–50)	2.5 (0–3.5)
SP-MS	5	100.0	60.0 (50–67) ^§^	5.5 (3.5–6.5)
PP-MS	2	50.0	63.5 (51–76) ^§^	6.5
Healthy controls	9	88.9	40.0 (27–55)	N/A
Samples used in Figure 5				
Total MS	11	90.9	45.0 (25–76)	3.5 (2.0–6.5)
RR-MS	7	85.7	31.0 (25–54)	3.5 (2.0–5.0)
SP-MS	1	100.0	51.0	4.0
PP-MS	3	100.0	46.0 (40–76)	5.5 (2.0–6.5)
Healthy controls	7	100.0	36.7 (27–55)	N/A

* RR-MS = relapsing-remitting MS; SP-MS = secondary progressive MS; PP-MS = primary progressive MS N/A= not applicable. ^§^ Significantly different (*P* < 0.05) to both RR-MS and Healthy control groups.

## Supplementary Materials

The following are available online at https://www.mdpi.com/2077-0383/9/8/2534/s1, Figure S1: Example of Western blots using cytoplasmic (C) and nuclear (N) fractions of PBMC, prepared using a NE-PERTM Nuclear and Cytoplasmic Extraction kit, and labelled with anti-PKCα antibody (specific for cytoplasm) or anti-PCNA antibody (specific for nucleus), to show that the cytosolic fractions did not contain nuclear material and that the nuclear fraction did not contain cytosolic proteins, respectively, and anti-actin antibody to show gel loading. HC = healthy control samples; MS = multiple sclerosis samples.
