# Supplementary material for: Reduced IκB-α Protein Levels in Peripheral Blood Cells of Patients with Multiple Sclerosis—A Possible Cause of Constitutive NF-κB Activation
Source: J Clin Med. 2020 Aug 6;9(8):2534. doi: 10.3390/jcm9082534 (PMC7465818; doi:10.3390/jcm9082534)

Supplementary Figure 1. Example of Western blots using cytoplasmic (C) and nuclear (N) fractions of PBMC, prepared using a NE-PERTM Nuclear and Cytoplasmic Extraction kit, and labelled with anti-PKCα antibody (specific for cytoplasm) or anti-PCNA antibody (specific for nucleus), to show that the cytosolic fractions did not contain nuclear material and that the nuclear fraction did not contain cytosolic proteins, respectively, and anti-actin antibody to show gel loading. HC = healthy control samples; MS = multiple sclerosis samples

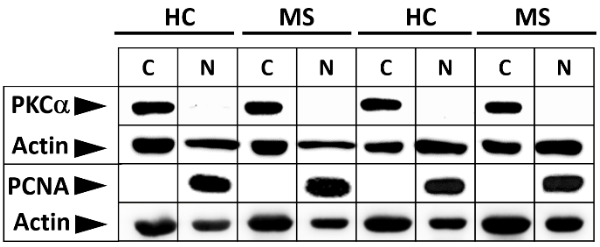

Supplement: Supplementary file 1 [file jcm-09-02534-s001.pdf]
